# Supplementary material for: Toxicity Impacts on Human Adipose Mesenchymal Stem/Stromal Cells Acutely Exposed to Aroclor and Non-Aroclor Mixtures of Polychlorinated Biphenyl
Source: Environ Sci Technol. 2023 Jan 18;57(4):1731–42. doi: 10.1021/acs.est.2c07281 (PMC9893815; doi:10.1021/acs.est.2c07281)
Supplement: Supplementary file 1 — es2c07281_si_001.pdf [file es2c07281_si_001.pdf]

Title: Toxicity impacts on human adipose MSCs acutely exposed to Aroclor and non-Aroclor mixtures of PCBs.

Authors
Riley M. Behan-Bush[1], Jesse N. Liszewski[1], Michael V. Schrodt, Bhavya Vats, Xueshu Li, Hans-Joachim Lehmler, Aloysius J. Klingelhutz, and James A. Ankrum[*]
[1]Contributed equally
*Corresponding Author: James-ankrum@uiowa.edu

Supplemental Information

Supplementary Figure 1: Adipose MSCs meet the ISCT Minimal Criteria.

Supplemental Figure 2: Exposure to PCB mixtures leads to dose dependent changes to MSC morphology.

Supplemental Table 1. qRT-PCR Primers

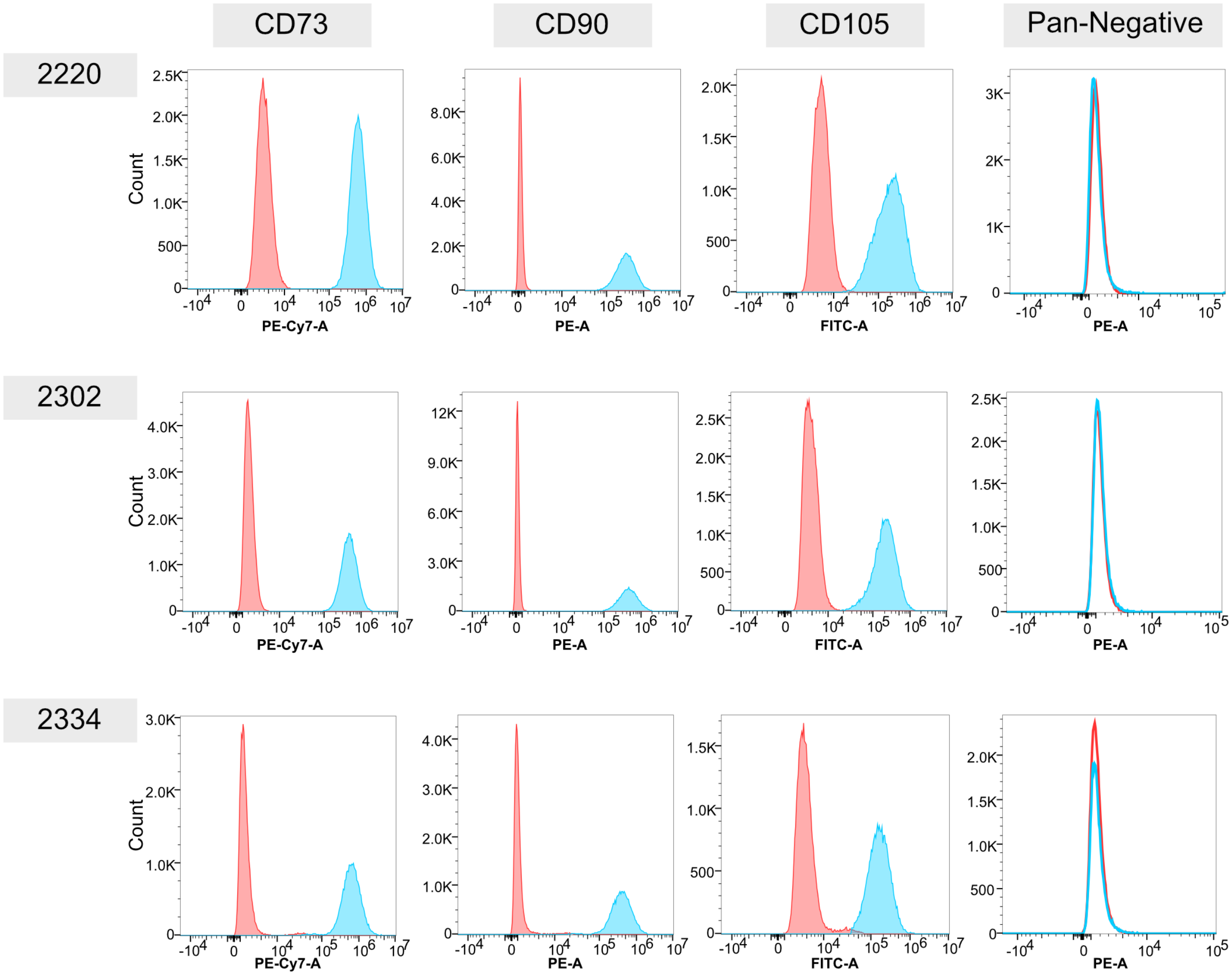

Donor	CD73	CD90	CD105	Negative
2220	100%	100%	99.80%	1.21%
2302	100%	100%	99.80%	0.71%
2334	99.50%	99.00%	98.20%	1.89%

Supplementary Figure 1: Adipose MSCs meet the ISCT Minimal Criteria. Adipose MSCs were analyzed for expression of markers according to the ISCT MSC Minimal Criteria. Flow cytometry histogram plots are shown for CD73, CD90, CD105 and a Negative Cocktail (CD34, CD45, CD11b, CD19, and HLA-DR). Each plot contains the isotype control (red peak) and on target sample (blue peak). Table displays the percent of each on-target population considered positive based on gates set using the isotype control.

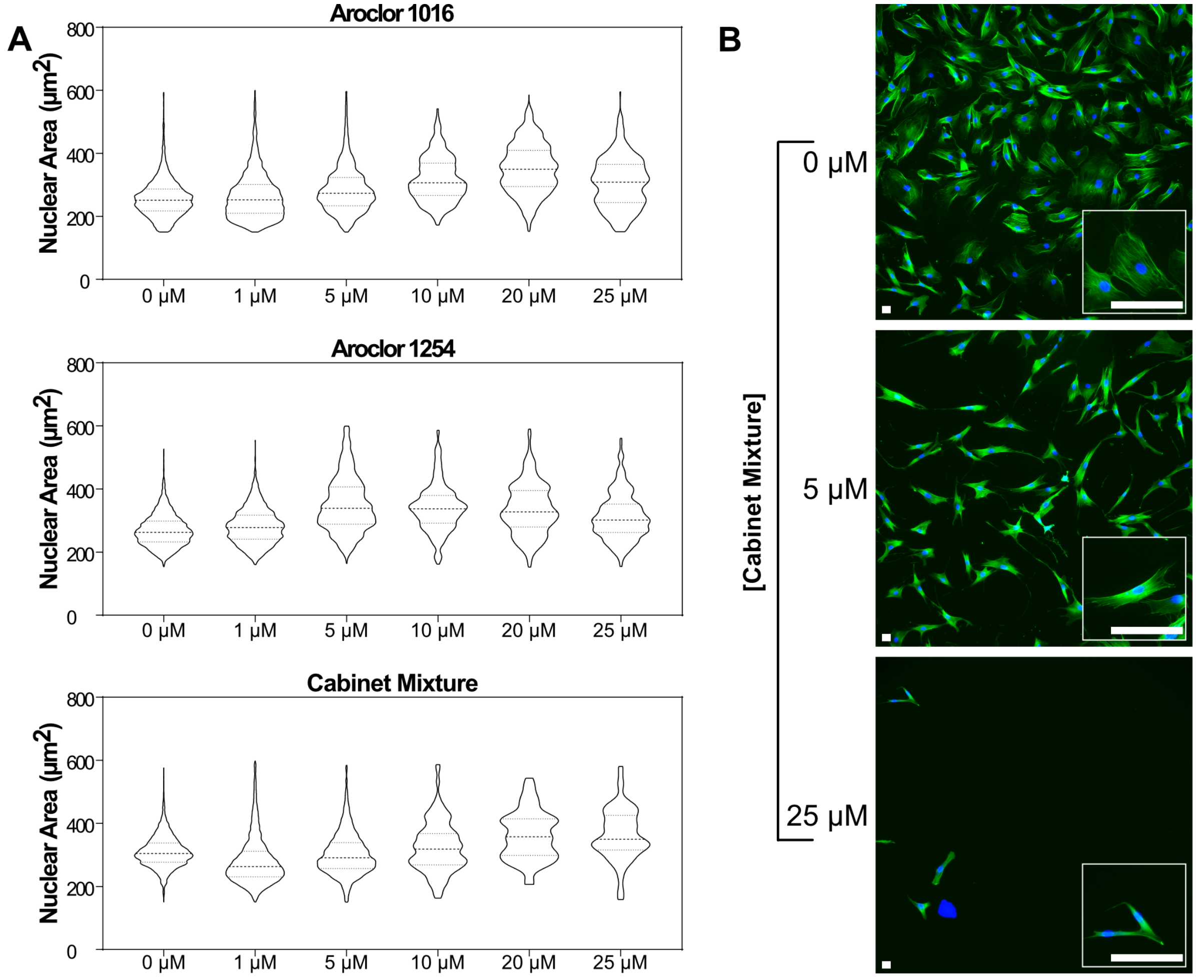

Supplemental Figure 2: Exposure to PCB mixtures leads to dose dependent changes to MSC morphology. Morphology of nuclei and cytoskeleton after 48 hours of exposure to PCB mixtures. (A) Violin plot of nuclear area after exposure to 0, 1, 5, 10, 20, or 25 µM concentrations of Aroclor 1016, Aroclor 1254, and Cabinet Mixture. (B) Representative images of cells after exposure to 0, 5, or 25 µM concentrations of Cabinet Mixture. All scale bars represent 50 µm.

Supplemental Table 1. qRT-PCR Primers

Primer Name; Exon Location	IDT Catalog #
GAPDH; 3 – 4	39a.22214836
ADIPOQ; 1 – 3	58.39189358
CCL2; 1 – 2	58.45467977
FABP6; 5 – 6	58.1261702
PPARG; 7 – 8	58.25464465
SCL2A4; 1 – 2	58.2557238
